# Clinical features and prognostic analysis of spontaneous rupture of renal cell carcinoma: a retrospective cohort study

**DOI:** 10.3389/fonc.2025.1598055

**Published:** 2025-10-10

**Authors:** Shanhua Zhang, Xiaoran Han, Shaoqiu Kong, Jianyu Wang, Cheng Wang, Fei Wu, Haihu Wu, Jiaju Lyu, Hao Ning

**Affiliations:** ^1^ Department of Urology, Shandong Provincial Hospital, Cheeloo College of Medicine, Shandong University, Jinan, Shandong, China; ^2^ Department of Urology, Shandong Provincial Hospital Affiliated to Shandong First Medical University, Jinan, China

**Keywords:** hemorrhage, Wunderlich syndrome, renal cell carcinoma, prognostic factors, spontaneous rupture, blood coagulation disorders

## Abstract

**Background:**

Spontaneous rupture of renal cell carcinoma (SRRCC) is a rare but critical clinical manifestation characterized by the acute disruption of tumor integrity, leading to extravasation of tumor contents into the perirenal space and associated complications. Understanding its clinical features, risk factors, and prognosis is essential for optimizing diagnosis and treatment.

**Methods:**

This retrospective, single-center study analyzed 37 cases of SRRCC from 165 patients with spontaneous renal tumor rupture treated between April 2014 and June 2024. Clinical, pathological, and laboratory data were collected and analyzed. Kaplan-Meier survival analysis and univariate Cox regression were used to evaluate cancer-specific survival (CSS) and progression-free survival (PFS).

**Results:**

The median age of SRRCC patients was 49 years (IQR: 38–60), with males accounting for 59.5%. Tumors were predominantly located in the left kidney (70.3%), with clear cell renal cell carcinoma (ccRCC) being the most common histological subtype (56.8%). The median tumor size was 6.5 cm(IQR: 4.1–10.1). The most frequent symptom was flank or abdominal pain (83.8%). Preoperative laboratory tests showed elevated APTT, PT, D-dimer, and fibrinogen levels. Radical nephrectomy was performed in 64.9% of patients, and 48.6% underwent emergency surgery. The median follow-up duration was 60 months (IQR: 27–80). The median cancer-specific survival (CSS) and progression-free survival (PFS) were 60 months (IQR: 27–80) and 49 months (IQR: 19–80), respectively. The 5-year CSS and PFS rates were 80.1% (95% CI: 64.0%–96.2%) and 68.8% (95% CI: 51.4%–86.2%), respectively, as estimated by the Kaplan–Meier method. Prognostic factors significantly associated with CSS and PFS included TNM stage, WHO/ISUP grade, tumor size, perirenal fat invasion, and coagulation abnormalities.

**Conclusions:**

SRRCC presents unique diagnostic and therapeutic challenges due to its acute nature and potential for tumor dissemination. Prognosis was associated with tumor characteristics and coagulation markers, while surgical timing and type was not significantly associated with outcomes. Further multicenter studies with larger cohorts are needed to validate these findings and guide clinical management.

## 1 Introduction

Spontaneous renal hemorrhage (SRH), also known as Wunderlich syndrome, is a rare but critical clinical condition characterized by acute bleeding within or around the kidney without preceding trauma or iatrogenic interventions (1). The most common symptom of SRH is the sudden onset of flank pain, often accompanied by hypotension, anemia, or shock in severe cases. Prompt recognition and management of SRH are crucial, as it can rapidly progress to life-threatening complications. Renal tumors are commonly associated with SRH, with angiomyolipoma (AML) being the most frequently reported pathology, followed by renal cell carcinoma (RCC). More recently, polyarteritis nodosa (PAN) has surpassed RCC as the second most common etiology of acute SRH, although RCC remains an important contributor to SRH cases (2).

However, spontaneous rupture of RCC (SRRCC) should be distinguished from SRH because of its unique pathophysiological mechanisms and clinical implications. Unlike SRH, which can arise from various benign and malignant etiologies, SRRCC specifically refers to the acute disruption of RCC integrity, leading to extravasation of tumor contents into the perirenal space and associated complications. This phenomenon is exceedingly rare and may result from increased internal tumor pressure, vascular invasion, or necrosis within the tumor microenvironment. Understanding these distinctions is essential for accurate diagnosis and management.

Current studies on SRRCC are limited to case reports, with systematic analyses rare due to the scarcity of large-scale clinical data. Investigating the clinical features, risk factors, and prognostic implications of RCC rupture is essential for optimizing treatment strategies, and enhancing patient outcomes. This study retrospectively analyzed 37 cases of spontaneous RCC rupture from a single center, systematically summarized their clinical characteristics, identified associated risk factors, and explored their associations with prognosis. The findings aim to provide valuable insights for clinical practice.

## 2 Materials and methods

### 2.1 Patient selection

This retrospective, single-center study analyzed data from 165 patients with spontaneous renal tumor rupture treated at a tertiary hospital between April 2014 and June 2024. Following screening, 37 patients who were postoperatively diagnosed with RCC were included in the final cohort. The inclusion criteria were as follows: ① preoperative imaging or intraoperative findings consistent with spontaneous renal tumor rupture. Specifically: (a) Imaging features included perirenal hematoma, contrast extravasation, or signs of renal capsule disruption on CT; (b) Intraoperative findings included visible rupture of tumor integrity, leakage of tumor contents into the perirenal space, or active bleeding with renal capsule laceration; ② postoperative pathological confirmation of RCC; ③ postoperative follow-up included computed tomography (CT) or other imaging studies to assess for recurrence or metastasis. The exclusion criteria were as follows: ① renal hemorrhage resulting from trauma or iatrogenic factors; ② preexisting renal conditions or a known history of tumor metastasis; ③ postoperative diagnosis of non-RCC renal lesions; and④ incomplete clinical data.

### 2.2 Data collection

Clinical data were collected from the electronic medical record system, outpatient visits, and telephone follow-ups and were categorized into several domains: general information, including age, sex, weight, smoking and alcohol history, underlying diseases (e.g., hypertension, diabetes, cardiovascular diseases), RENAL score, and ASA classification; clinical symptoms, such as flank pain, abdominal pain, fever, nausea and vomiting, and hematuria; laboratory and imaging data, including preoperative laboratory test results and imaging findings; pathological data, including pathological type, tumor size, TNM stage, WHO/ISUP grade, tumor necrosis, and perirenal fat invasion; and follow-up data, including disease progression events such as local recurrence, distant metastasis, or cancer-related death. This study was approved by the Ethics Committee of the hospital, and informed consent was obtained from all patients.

The primary outcome measures included cancer-specific survival (CSS) and progression-free survival (PFS). CSS was defined as the time from surgery to death specifically attributed to renal cell carcinoma. PFS was defined as the time from surgery to any event of disease progression, including local recurrence, distant metastasis, or cancer-related death. The secondary outcome measures included surgery-related factors, such as the type of surgery (radical nephrectomy or partial nephrectomy), timing of surgery (immediate or delayed), and surgical waiting time (the interval from diagnosis to surgery). The clinical characteristics assessed included the prevalence of preoperative symptoms such as flank pain, hematuria, nausea, vomiting, and shock. The laboratory test data included preoperative coagulation markers (e.g., prothrombin time, activated partial thromboplastin time, D-dimer, and fibrinogen levels) and other routine blood parameters. The pathological features included tumor size, TNM stage, WHO/ISUP grade, tumor necrosis, and perinephric fat invasion.

Patients with missing follow-up data were included in descriptive and baseline analyses but excluded from survival analysis.We handled right-censored data using Kaplan-Meier survival analysis techniques.The final survival analysis included only patients with complete follow-up information on survival status and time-to-event outcomes.

Notably, none of the patients in this cohort received adjuvant therapy during the follow-up period. Postoperative evaluations were routinely performed using abdominal ultrasound, chest CT or X-ray, and abdominal CT to assess for recurrence or metastasis.

### 2.3 Ethical considerations

This study was approved by the Ethics Committee of Shandong Provincial Hospital (Shandong Provincial Hospital Biomedical Research Ethics Committee Involving Human Subjects, Approval Number: SWYX: NO.2025-006). Informed consent was waived due to the retrospective nature of the study and the use of anonymized clinical data. The research was conducted in accordance with the Declaration of Helsinki and relevant local regulations. The data used in this study were accessed for research purposes on June 3, 2024. Although researchers had access to identifiable information during data collection, all necessary safeguards were implemented to protect participant confidentiality, in compliance with ethical standards.

### 2.4 Statistical methods

Data analysis was conducted using SPSS version 27.0. The normality of continuous variables was assessed using the Shapiro–Wilk test. Variables are reported as medians and interquartile ranges (IQRs). Categorical variables are expressed as frequencies and percentages (n, %).Receiver operating characteristic (ROC) curve analysis was performed to determine optimal cutoff points of continuous variables for predicting survival outcomes. CSS and PFS were analyzed using the Kaplan–Meier method. The median follow-up duration was calculated using the reverse Kaplan–Meier method and is presented as median [IQR]. Median CSS and PFS times, as well as 5-year survival rates, were estimated with corresponding 95% confidence intervals. Differences between survival curves were evaluated using the log-rank test.In addition, univariate Cox proportional hazards regression analysis was conducted to examine the associations between clinical or pathological factors and survival outcomes. Results were expressed as hazard ratios (HRs) with 95% confidence intervals (CIs). Statistical significance was defined as P < 0.05.

## 3 Results

### 3.1 General and surgical characteristics

The baseline characteristics and surgical details of the 37 patients are summarized in **Table 1**. The median age of SRRCC patients was 49 years (IQR: 38–60), and the majority were male (59.5%). The comorbidities included hypertension (24.3%), diabetes (13.5%), and cardiovascular diseases (10.8%). The tumors were predominantly located in the left kidney (70.3%) and most commonly in the upper pole (45.9%). The median tumor size was 6.5 cm (IQR: 4.1–10.1).

**Table 1:** General and surgical characteristics of patients.

Character	No. (percentage) Median [IQR]
Age (years)	49 [38,60]
Gender	
Male	22(59.5%)
Female	15(40.5%)
Weight (kg)	71 [64,80]
Smoking	12(32.4%)
Drinking	13(35.1%)
Hypertension	9(24.3%)
Diabetes	5(13.5%)
CVDs	4(10.8%)
History of abdominal surgery	7(18.9%)
Flank/Abdominal Pain	31(83.8%)
Fever	1(2.7%)
Nausea/Vomiting	3(8.1%)
Hematuria	6(16.2%)
Asymptomatic	3(8.1%)
Shock	1(2.7%)
Tumor Diameter (cm)	6.5 [4.1,10.1]
Tumor Location	
Upper Pole	17(45.9%)
Middle Pole	8(21.6%)
Lower Pole	12(32.4%)
Tumor Side	
Left	26(70.3%)
Right	11(29.7%)
Onset Days	102 [8,65]
Surgical Approach	
Open	6(16.2%)
Laparoscopic	28(75.7%)
Robot-assisted	3(8.1%)
Surgical Type	
Partial Nephrectomy	13(35.1%)
Radical Nephrectomy	24(64.9%)
Surgical Timing	
Immediate Surgery	18(48.6%)
Conservative Surgery	17(45.9%)
Embolization Surgery	2(5.4%)
ASA	
II	31(83.8%)
III	6(16.2%)
RENAL	
6	5(13.5%)
7	22(59.5%)
8	5(13.5%)
9	3(8.1%)
10	2(5.4%)
Surgical Time (minutes)	180 [137,223]
Transfusion Rate (%)	3(8.1%)
Drainage Volume (mL)	350 [110,370]
Drain Removal Time (days)	5[3,6]
Intraoperative Blood Loss (mL)	188 [50,300]
Preoperative Neutrophil Count(10^9^/L)	5.6 [3.4,9.4]
Preoperative Lymphocyte Count(10^9^/L)	1.4 [0.9,1.8]
Preoperative Urea Nitrogen(mmol/L)	5.0 [4.4,5.9]
Preoperative APTT(s)	31.5 [27.7,34.5]
Preoperative PT(s)	11.9 [11.4,12.9]
Preoperative Hemoglobin (g/L)	131 [111,144]
Preoperative Platelet Count (10^9^/L)	234 [184,294]
Preoperative Alkaline Phosphatase (U/L)	83 [70,98]
Preoperative Calcium (mmol/L)	2.3 [2.2,2.4]
Preoperative Potassium (mmol/L)	4.1 [3.9,4.4]
Preoperative Sodium (mmol/L)	140.4 [138.0,141.5]
Preoperative D-Dimer (µg/mL)	1.3 [0.2,1.9]
Preoperative Fibrinogen(g/L)	3.7 [2.6,4.3]
Preoperative NLR	7.3 [1.9,7.6]
Preoperative Cystatin C(mg/L)	1.0 [0.8,1.1]
Preoperative Creatinine(µmol/L)	71.7 [57.9,76.6]
Preoperative Albumin(g/L)	41.0 [38.2,43.5]

The most common symptom was flank or abdominal pain (83.8%), whereas other symptoms such as hematuria, nausea, vomiting, and fever were less common. One patient presented with shock, and three patients were diagnosed incidentally.

Laparoscopic surgery was the predominant approach (75.7%), and radical nephrectomy was performed in 64.9% of patients. With respect to surgical timing, 48.6% of the patients underwent immediate surgery, whereas the remaining patients underwent delayed interventions. The majority of patients were classified as ASA II (83.8%).

Preoperative laboratory tests revealed stable coagulation function. The median activated partial thromboplastin time (APTT) and prothrombin time (PT) were 31.5 seconds (IQR: 27.7–34.5) and 11.9 seconds (IQR: 11.4–12.9), respectively. The median D-dimer level was 1.3 µg/mL (IQR: 0.2–1.9), and fibrinogen was 3.7 g/L (IQR: 2.6–4.3), indicating no major preoperative coagulopathy among the cohort. Operative metrics, such as the median drain output and drain removal time, are detailed in **Table 1**.

To reduce the potential for selection bias, baseline characteristics were compared between groups stratified by TNM stage and surgical timing. Due to the small subgroup sizes, T2 and T3 stages were combined and compared with T1 tumors, while patients who underwent surgery after embolization or conservative management were grouped together and compared with those who received emergency surgery. No significant differences in baseline characteristics were observed between the surgical timing groups (**Supplementary Table S1**). For TNM staging, compared with the T1 group, the higher-stage group (T2–T3) had a significantly larger tumor diameter, a higher frequency of gross hematuria, and lower preoperative hemoglobin levels, while other variables showed no significant differences (**Supplementary Table S2**).

### 3.2 Pathological and follow-up data

Postoperative pathology revealed that clear cell renal cell carcinoma (ccRCC) was the predominant histological subtype, accounting for 56.8% of cases. Other subtypes included chromophobe renal cell carcinoma (16.2%), papillary renal cell carcinoma (13.5%), genetic-associated renal carcinoma (10.8%), and eosinophilic renal carcinoma (2.7%). Tumor grading and staging indicated that the majority of patients were classified as WHO/ISUP grade II (59.5%), whereas 24.3% of tumors were staged as T1a according to the TNM system. Additionally, tumor necrosis was observed in 13.5% of the patients, and perinephric fat invasion occurred in 5.4%.

Over a median follow-up of 60 months (IQR: 27–80), 6 patients (16.2%) died from RCC, and 10 patients (27%) experienced disease progression, including local progression or distant metastasis. The median CSS and PFS were 60 months (IQR: 27–80) and 49 months (IQR: 19–80), respectively. Two patients (5.4%) were lost to follow-up. Postoperative management, such as systemic anticancer therapy, was reserved for patients with recurrence or metastasis. A detailed summary of the pathological findings and clinical outcomes is presented in **Table 2**.

**Table 2:** Pathological and follow-up data of patients.

Character	No. (percentage) Median [IQR]
Histological subtypes	
Papillary Renal Cell Carcinoma	5(13.5%)
Eosinophilic Renal Cell Carcinoma	1(2.7%)
Clear Cell Carcinoma	21(56.8%)
Chromophobe Renal Cell Carcinoma	6(16.2%)
Genetic Associated Renal Cell Carcinoma	4(10.8%)
TNM staging	
T1a	9(24.3%)
T1b	10(27.0%)
T2a	6(16.2%)
T2b	9(24.3%)
T3a	3(8.1%)
WHO/ISUP grading	
Grade I	8(21.6%)
Grade II	22(59.5%)
Grade III	7(18.9%)
Metastasis	6(16.2%)
Local recurrence	4(10.8%)
Death	6(16.2%)
Perinephric Fat Invasion	2(5.4%)
Tumor Necrosis	5(13.5%)
CSS	60 [27,80]
PFS	49 [19,80]
Lost to Follow-up	2(5.4%)

### 3.3 Survival analysis

During a median follow-up of 60 months (IQR: 27–80), 7 patients developed distant metastasis. The most common sites of metastasis were the liver (3 patients, 42.9%) and the lungs (3 patients, 42.9%). Other metastatic sites included the adrenal gland (1 patient, 16.7%) and bone (1 patient, 16.7%), with one patient having both bone and liver metastases simultaneously. Local recurrence occurred in 3 patients, and 6 patients died during the follow-up period.

In this cohort, the 5-year CSS rate for patients with SRRCC was 80.1%(95% CI: 64.0%–96.2%), while the 5-year PFS rate was 68.8% (95% CI: 51.4%–86.2%). Kaplan-Meier survival analysis revealed no significant differences in oncological outcomes, including CSS and PFS, between radical nephrectomy and partial nephrectomy.

**Figure 1:**
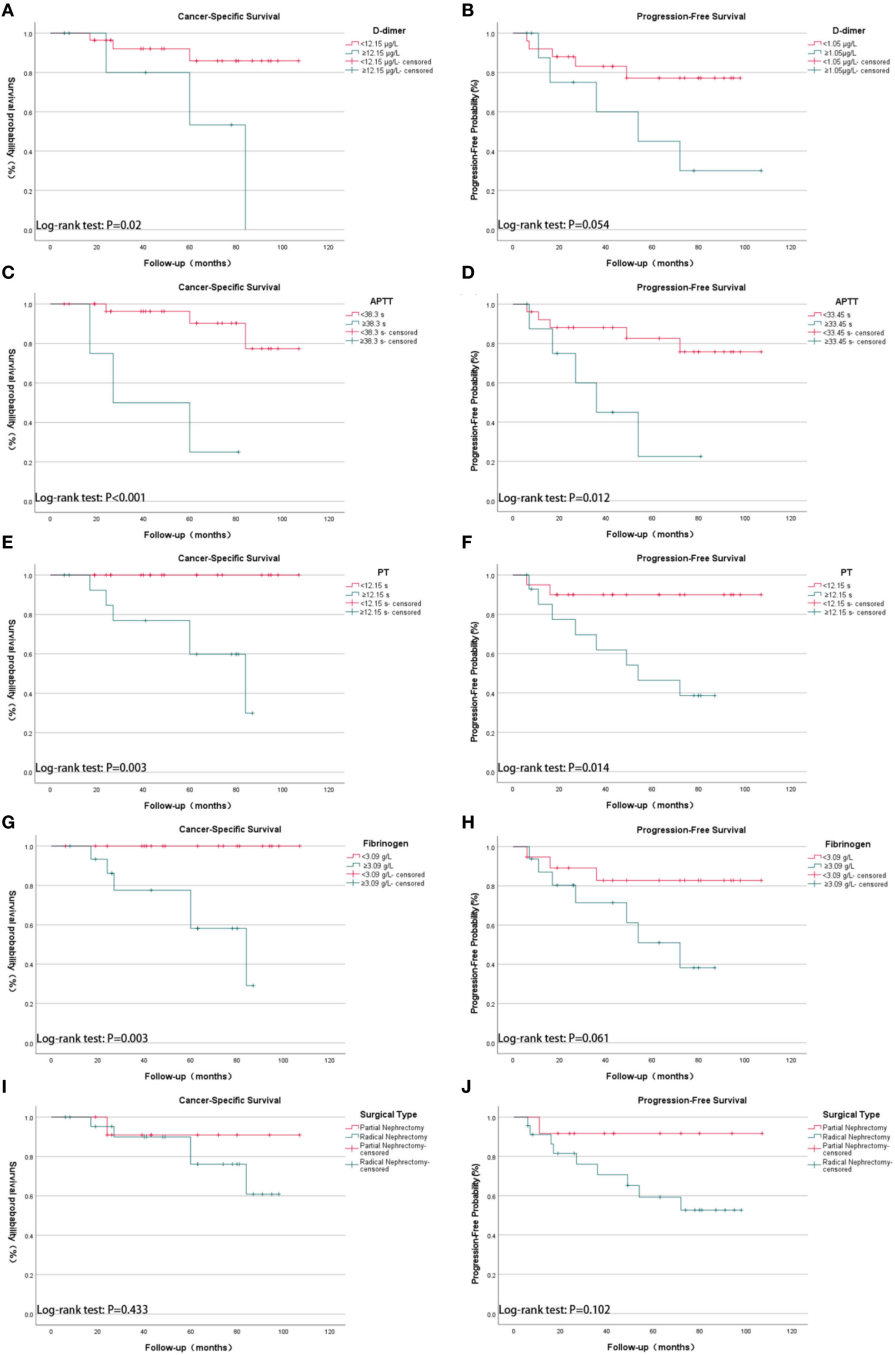
Kaplan-Meier survival analysis of cancer-specific survival (CSS) and progression-free survival (PFS) in 35 patients with spontaneous rupture of renal cell carcinoma (SRRCC), stratified by coagulation parameters and surgical types. **(A)** CSS by D-dimer levels: <12.15 µg/mL vs. ≥12.15 µg/mL (p = 0.02); **(B)** PFS by D-dimer levels: <12.15 µg/mL vs. ≥12.15 µg/mL (p = 0.054); **(C)** CSS by activated partial thromboplastin time (APTT): <38.3 s vs. ≥38.3 s (p < 0.001); **(D)** PFS by APTT: <38.3 s vs. ≥38.3 s (p = 0.012); **(E)** CSS by prothrombin time (PT): <12.15 s vs. ≥12.15 s (p = 0.003); **(F)** PFS by PT: <12.15 s vs. ≥12.15 s (p = 0.014); **(G)** CSS by fibrinogen levels: <3.09 g/L vs. ≥3.09 g/L (p = 0.003); **(H)** PFS by fibrinogen levels: <3.09 g/L vs. ≥3.09 g/L (p = 0.061); **(I)** CSS by surgical type: partial nephrectomy vs. radical nephrectomy (p = 0.433); **(J)** PFS by surgical type: partial nephrectomy vs. radical nephrectomy (p = 0.102). The y-axis represents survival probability (%) for CSS plots and progression-free probability (%) for PFS plots; the x-axis represents follow-up time in months. Groupings were based on optimal cutoff values determined by ROC analysis.

Kaplan–Meier survival analysis (log-rank test) demonstrated that several factors were significantly associated with CSS, including TNM stage, WHO/ISUP grade, age, tumor size, cardiovascular diseases, PT, APTT, perinephric fat invasion, D-dimer, and fibrinogen levels. For PFS, significant associations were observed with TNM stage, WHO/ISUP grade, tumor size, PT, APTT, and perinephric fat invasion (**Figure 1**).

Univariate Cox regression analysis further revealed that TNM stage, WHO/ISUP grade, cardiovascular diseases, PT, APTT, RENAL score, perinephric fat invasion, D-dimer, and fibrinogen were significantly associated with CSS. Prognostic factors significantly associated with PFS included TNM stage, WHO/ISUP grade, tumor size, cardiovascular diseases, and coagulation abnormalities (**Table 3**).

**Table 3:** Univariate Cox proportional hazards analysis for CSS and PFS.

Variable	CSS			PFS		
	HR	95% CI of HR	P value	HR	95% CI of HR	P value
Perinephric Fat Invasion	30.463	1.904-487.438	0.016	9.708	0.861-109.419	0.066
Tumor Necrosis	0.312	0.034-2.896	0.306	1.332	0.164-10.841	0.788
TNM staging						
T3			0.021			0.027
T1	0.026	0.002-0.355	0.006	0.23	0.078-0.684	0.008
T2	0.188	0.03-1.197	0.077	1.24	0.529-2.907	0.621
WHO/ISUP grading						
Grade III			0.034			0.001
Grade I	0.096	0.007-1.293	0.077	0.063	0.007-0.579	0.015
Grade II	0.038	0.003-0.504	0.013	0.077	0.017-0.355	0.001
Surgical Timing	1.648	0.399-6.806	0.49	2.745	0.772-9.766	0.119
Surgical Approach	0.313	0.056-1.739	0.184	0.359	0.103-1.25	0.108
Surgical Type	2.291	0.266-19.703	0.45	4.763	0.602-37.668	0.139
Age (years)	1.045	0.981-1.114	0.173	1.018	0.972-1.066	0.444
Tumor Diameter(cm)	1.207	0.99-1.47	0.062	1.169	1.01-1.353	0.036
Onset Days(days)	1	0.995-1.004	0.888	2.962	0.762-11.52	0.117
Cardiovascular diseases	9.955	1.644-60.292	0.012	1.791	1.07-2.998	0.027
RENAL	2.15	1.069-4.325	0.032	1.13	0.996-1.282	0.058
Preoperative APTT(s)	1.229	1.021-1.48	0.029	1.615	1.019-2.56	0.041
Preoperative PT(s)	2.084	1.179-3.687	0.012	1.03	0.613-1.73	0.911
Histological Subtypes	0.525	0.175-1.571	0.249	1.001	1-1.002	0.106
Preoperative Fibrinogen(g/L)	2.331	1.297-4.19	0.005	1.405	0.9115-2.157	0.12
Preoperative D-Dimer (µg/mL)	1.747	1.013-3.01	0.045	1.917	1.199-3.068	0.007

## 4 Discussion

SRRCC is a rare but severe clinical manifestation with pathophysiological mechanisms and clinical implications that are significantly different from those of other forms of SRH. Although the overall incidence of SRH is less than 1%, RCC is frequently associated with tumor-related SRH. Studies by Daskalopoulos et al. reported that 30% of SRH cases were associated with RCC (3), whereas Zhang et al. reported that 26.1% of SRH cases were also associated with RCC (4).The occurrence of SRRCC may result from increased internal tumor pressure, vascular wall fragility, or tumor necrosis, presenting unique challenges in terms of diagnosis, treatment, and prognosis (5, 6).

In this study, ccRCC was the most common histological subtype, accounting for 56.8% of all cases. Its unique molecular and pathological characteristics make it particularly prone to spontaneous rupture. Specifically, the inactivation of the Von Hippel–Lindau (VHL) tumor suppressor gene in ccRCC leads to the accumulation of hypoxia-inducible factors (HIFs), which in turn upregulate pro-angiogenic factors such as vascular endothelial growth factor (VEGF). This cascade promotes the development of abnormal, fragile, and highly permeable neovascular networks within the tumor. These structurally immature vessels, combined with increased intratumoral pressure and areas of necrosis, significantly increase the risk of spontaneous hemorrhage and capsular disruption, thereby contributing to tumor rupture (7–9). Our study further indicated that the biological characteristics of tumors were significantly associated with the prognosis of patients with spontaneous RCC rupture. Factors such as tumor stage, grade, and maximum tumor diameter were found to be significantly associated with prognosis. Notably, although ruptures are more common in T1a to T2b tumors, smaller or more localized tumors can also rupture under certain conditions. This finding indicates that tumor rupture is not solely related to larger or more advanced tumors but may also be influenced by other pathophysiological mechanisms, such as changes in local vascular pressure or abnormalities in the tumor microenvironment. Understanding these mechanisms is crucial for further optimizing the diagnosis and management of SRRCC.

In contrast to patients with SRRCC, those with non-ruptured RCC generally have more favorable long-term oncological outcomes. Bradshaw et al. reported 5-year overall survival (OS) and disease-free survival rates of 76.3% and 78.6%, respectively, in a cohort of 648 patients with cT2a RCC (10). Likewise, Amparore et al. observed a 5-year PFS rate of 92.2% among 116 patients with cT2 RCC (11). In a study focusing on cT3a RCC, Yim et al. demonstrated 5-year recurrence-free survival and CSS rates of 82.1% and 93.3%, respectively, underscoring the relatively favorable prognosis in non-ruptured RCC (12). By contrast, our study revealed substantially poorer outcomes in patients with SRRCC, with 5-year CSS and PFS rates of 80.1% and 68.8%, respectively. These findings indicate that tumor rupture may be associated with adverse prognosis; however, whether it serves as a risk factor for adverse prognosis requires further investigation.

Moreover, perinephric fat invasion was identified as a prognostic factor significantly associated with CSS. Campbell et al. similarly reported that tumor rupture extending beyond Gerota’s fascia was associated with poor prognosis (13). Okada et al. reported that in patients with spontaneous rupture of renal cancer, those with hemorrhage extending beyond Gerota’s fascia exhibited a lower cumulative survival rate. However, statistical analysis using the Wilcoxon test showed no significant difference, suggesting a potential impact on overall prognosis without statistical significance (14). In the present study, only two patients exhibited perinephric fat invasion, and thus, no formal statistical analysis was conducted for this variable. Among them, one patient developed distant metastasis and died due to tumor progression, while the other had a follow-up period of only 8 months and showed no adverse outcomes. In comparison, among the remaining 33 patients without perinephric invasion, five deaths were recorded. Given the limited sample size and the small number of events within this subgroup, definitive conclusions could not be drawn. Nevertheless, in the context of SRRCC, rupture extending beyond Gerota’s fascia remains particularly concerning, as it may increase the risk of tumor dissemination and worsen clinical outcomes. When rupture extends beyond Gerota’s fascia, the resulting hematoma may obscure tumor margins, complicate radiological interpretation, interfere with surgical planning, and ultimately contribute to poorer clinical outcomes.

This study found that prolonged APTT and PT, along with elevated levels of D-dimer and fibrinogen, were significantly associated with poorer CSS. Coagulation abnormalities observed in SRRCC may be linked to disease progression through multiple underlying mechanisms. Notably, these abnormalities are not merely passive responses to tumor rupture, but may also actively contribute to tumorigenesis and progression.

Extensive evidence suggests that tumor cells can activate the coagulation cascade by secreting tissue factor (TF) and various pro-inflammatory cytokines, thereby inducing a hypercoagulable state. TF, a key initiator of the extrinsic coagulation pathway, is upregulated in many malignancies, including RCC, and plays a critical role in tumor proliferation, invasion, and metastasis. TF-mediated thrombin generation further activates protease-activated receptors, which promote tumor cell growth, angiogenesis, and epithelial–mesenchymal transition. Additionally, tumor-derived microvesicles can activate platelets, which in turn release growth factors such as TGF-β and VEGF, contributing to the remodeling of the tumor microenvironment and facilitating immune evasion and neovascularization. Activated platelets may also form a physical shield around tumor cells, protecting them from natural killer cell-mediated clearance and promoting distant metastasis (15, 16).This TF- and platelet-driven hypercoagulable state not only reflects enhanced tumor aggressiveness but also provides a mechanistic rationale for the prognostic significance of coagulation markers in SRRCC and highlights the potential role of anticoagulant strategies as adjunctive therapeutic approaches.

The treatment options for SRRCC include conservative management, selective arterial embolization (SAE), and emergency surgery. In this study, emergency surgery was the most common treatment (48.6%), followed by conservative management (45.9%) and embolization (5.4%). Given the potentially life-threatening nature of SRRCC in hemodynamically unstable patients, prompt emergency surgical intervention is crucial (17). Although our study did not reveal statistically significant differences in survival among different treatment strategies, the risk of delayed diagnosis and definitive management of malignancies associated with conservative treatment should not be underestimated. Preoperative imaging often fails to reliably differentiate benign from malignant renal masses (e.g., AML vs. RCC), which may delay appropriate intervention. Some researchers recommend early radical nephrectomy in cases of spontaneous perirenal hemorrhage with uncertain etiology to avoid missing an undiagnosed RCC (18, 19). while others advocate for close radiological follow-up with CT until the hematoma resolves and a definitive diagnosis can be made (20, 21). In our study, the average waiting time for patients who underwent direct surgery was 9 ± 4 days; for those who underwent surgery after conservative management, the waiting time was 205 ± 444 days; and for those who underwent surgery after embolization, the waiting time was 141 ± 187 days. Kaplan–Meier analysis indicated that surgical waiting time was not significantly associated with survival prognosis, but optimizing the timing of surgery remains an area for further investigation.

Kaplan–Meier analysis revealed no statistically significant differences in CSS or PFS between patients who underwent radical nephrectomy and those who underwent partial nephrectomy. However, this result should be interpreted with caution, as the proportion of patients receiving partial nephrectomy was relatively low (35.1%), which may have limited the statistical power to detect differences.Previous studies have suggested that patients undergoing partial nephrectomy typically have smaller tumors and lower-stage disease, which may account for similar oncological outcomes between the two approaches (22).In our cohort, partial nephrectomy cases did not show a clear advantage in tumor size or stage, supporting its feasibility as a kidney-sparing option when appropriate. Nevertheless, for SRRCC patients, radical nephrectomy remains the preferred approach in many cases due to its effectiveness in controlling both the tumor and hemorrhage, especially in larger or more aggressive tumors.

In summary, coagulation abnormalities, including prolonged PT and APTT, along with elevated D-dimer and fibrinogen levels, were observed in patients with poorer prognosis. However, no statistically significant differences in CSS or PFS were observed between different treatment modalities and surgical types. Further studies with larger sample sizes and multicenter data are needed to determine the optimal timing and approach for surgery, as well as to investigate the role of coagulation abnormalities in the progression of SRRCC.

Moreover, due to the lack of data on adjuvant therapy in patients with SRRCC, its specific efficacy remains unclear. Adjuvant therapy is defined as systemic treatment administered after complete tumor resection, in the absence of detectable disease on postoperative imaging, to reduce recurrence risk. In this retrospective cohort, systemic therapy was administered only upon disease recurrence or metastasis, which was consistent with the standard of care during the study period.

In the era of targeted therapy, four randomized controlled trials (ASSURE, SORCE, S-TRAC, and PROTECT) investigated the efficacy of adjuvant targeted agents but produced inconsistent results, with no definitive survival benefit observed (23–25). More recently, based on the results of the KEYNOTE-564 trial, pembrolizumab has been approved as adjuvant therapy for patients with clear cell RCC at intermediate-high or high risk of recurrence after nephrectomy, or those who have undergone complete resection of metastases within 12 months, with the aim of improving survival outcomes. This indication is derived from clinical trials with specific cohort characteristics (26, 27).

However, the patients in this study were treated prior to the implementation of pembrolizumab as a standard adjuvant option. Additionally, SRRCC represents a unique clinical scenario not specifically addressed in these trials. Tumor rupture may profoundly alter the tumor microenvironment, potentially influencing therapeutic response. Therefore, the role of adjuvant therapies, including targeted and immune-based treatments, in SRRCC remains to be further explored. This study aims to provide preliminary evidence for guiding clinical decision-making in this distinct subset of RCC patients.

This study has several limitations. First, as a single-center retrospective analysis with a limited sample size (n = 37), multivariate Cox regression analysis was not feasible due to the risk of model overfitting and unstable estimates. Therefore, only univariate analyses were performed, which may limit the ability to control for potential confounding or covariate variables such as age, comorbidities, and tumor burden, reducing the statistical power and generalizability of the findings. Second, the follow-up duration varied across patients, with some cases having relatively short follow-up periods, which may affect the accuracy of long-term survival estimation. Third, this study primarily relied on clinical and pathological data and did not investigate the molecular mechanisms underlying coagulation abnormalities and tumor rupture, such as VHL gene mutations or dysregulation of the HIFs pathway, which have been implicated in the pathogenesis and progression of clear cell RCC. The absence of these data limits the depth of mechanistic understanding and precludes analysis of potential molecular predictors of tumor rupture. Future research should involve multicenter, large-scale prospective cohorts and incorporate molecular analyses to validate and expand upon our findings.

## 5 Conclusions

SRRCC is a rare but significant clinical manifestation characterized primarily by flank or abdominal pain, with a minority of patients presenting with shock or being diagnosed incidentally. Our study showed that SRRCC predominantly occurs in middle-aged men, with a median tumor diameter of 6.5 cm(IQR: 4.1–10.1), and is most commonly located in the left kidney. ccRCC is the most common histological subtype. The prognosis of SRRCC patients is associated with various factors, including TNM stage, WHO/ISUP grade, tumor size, perirenal fat invasion, and coagulation abnormalities. The median CSS and PFS were 60 months (IQR: 27–80) and 49 months (IQR: 19–80), respectively, as estimated by Kaplan-Meier analysis. Elevated preoperative levels of APTT, PT, D-dimer, and fibrinogen suggest a tumor-related hypercoagulable state that may be associated with disease progression. Furthermore, no statistically significant associations were observed between the type or timing of surgery and survival outcomes in this cohort. Although this study provides preliminary insights into the clinical characteristics and prognostic patterns of SRRCC, which warrant validation in larger prospective studies.

## Data Availability

The datasets generated and/or analyzed during the current study are not publicly available due to the use of patient data and the need to protect patient privacy. However, the datasets are available from the corresponding author upon reasonable request, subject to ethical and legal considerations. Requests to access the datasets should be directed to Zhang Shanhua zsh15153206664@163.com.

## Supplementary material

Supplementary Table S1 Baseline characteristics by surgical timing.

Supplementary Table S2 Baseline characteristics by TNM stage.
