# Supplementary material for: Clinical features and prognostic analysis of spontaneous rupture of renal cell carcinoma: a retrospective cohort study
Source: Front Oncol. 2025 Oct 10;15:1598055. doi: 10.3389/fonc.2025.1598055 (PMC12549311; doi:10.3389/fonc.2025.1598055)
Supplement: Supplementary Table S1 — Baseline characteristics by surgical timing. [file Table1.docx]

Baseline characteristics by surgical timing

| Preoperative Characteristics | Emergency Surgery  (n=17), Median [IQR] | Delayed Surgery (n=18), Median [IQR] | P-value |
| --- | --- | --- | --- |
| Age (years) | 50 [37,66] | 49 [42,54] | 0.891 |
| Gender |  |  | 0.118 |
| Male | 8 | 12 |  |
| Female | 10 | 5 |  |
| Weight (kg) | 69 [63,85] | 73 [64,80] | 0.637 |
| Smoking | 5 | 5 | 0.915 |
| Drinking | 4 | 7 | 0.227 |
| Hypertension | 2 | 6 | 0.529 |
| Diabetes | 1 | 3 | 0.678 |
| CVDs | 0 | 4 | 0.125 |
| History of abdominal surgery | 1 | 5 | 0.061 |
| Flank/Abdominal pain | 13 | 16 | 0.086 |
| Fever | 1 | 0 | 0.324 |
| Nausea/Vomiting | 1 | 1 | 0.967 |
| Hematuria | 5 | 1 | 0.086 |
| Shock | 0 | 1 | 0.464 |
| Tumor Diameter(cm) | 6.4 [4.6,10.4] | 4.9 [3.4,9.2] | 0.335 |
| Tumor location |  |  | 0.666 |
| Upper pole | 9 | 7 |  |
| Middle pole | 3 | 5 |  |
| Lower pole | 6 | 5 |  |
| Tumor Side |  |  | 0.328 |
| Left | 11 | 13 |  |
| Right | 7 | 4 |  |
| Preoperative creatinine (µmol/L) | 69.6 [53.6,74.8] | 71.8 [63.5,80.6] | 0.520 |
| Preoperative hemoglobin (g/L) | 125 [105,135] | 139 [124,146] | 0.093 |
