# Supplementary material for: Clinical features and prognostic analysis of spontaneous rupture of renal cell carcinoma: a retrospective cohort study
Source: Front Oncol. 2025 Oct 10;15:1598055. doi: 10.3389/fonc.2025.1598055 (PMC12549311; doi:10.3389/fonc.2025.1598055)
Supplement: Supplementary Table S2 — Baseline characteristics by TNM stage. [file Table2.docx]

Baseline characteristics by TNM stage

| Preoperative Characteristics | Low-stage (n=19),Median [IQR] | High-stage (n=16),Median [IQR] | P-value |
| --- | --- | --- | --- |
| Age (years) | 48 [44,60] | 50 [38,59] | 0.868 |
| Gender |  |  | 0.268 |
| Male | 13 | 8 |  |
| Female | 6 | 8 |  |
| Weight (kg) | 70 [63,80] | 72 [62,80] | 0.740 |
| Smoking | 6 | 5 | 0.983 |
| Drinking | 6 | 6 | 0.713 |
| Hypertension | 5 | 3 | 0.595 |
| Diabetes | 3 | 1 | 0.377 |
| CVDs | 1 | 3 | 0.212 |
| History of abdominal surgery | 2 | 4 | 0.258 |
| Flank/Abdominal pain | 16 | 13 | 0.817 |
| Fever | 0 | 1 | 0.269 |
| Nausea/Vomiting | 0 | 2 | 0.112 |
| Hematuria | 1 | 5 | 0.042 |
| Shock | 0 | 1 | 0.269 |
| Tumor Diameter(cm) | 4.1 [3.4,5.6] | 10.1 [8.4,12.3] | <0.001 |
| Tumor location |  |  | 0.243 |
| Upper pole | 9 | 7 |  |
| Middle pole | 4 | 7 |  |
| Lower pole | 6 | 2 |  |
| Tumor Side |  |  | 0.983 |
| Left | 6 | 5 |  |
| Right | 13 | 11 |  |
| Preoperative creatinine (µmol/L) | 66.0 [55.0,76.2] | 73.2 [66.8,79.1] | 0.164 |
| Preoperative hemoglobin (g/L) | 135 [127,148] | 116 [105,137] | 0.021 |
